# Cardiovascular Risks Associated with Low Dose Ionizing Particle Radiation

**DOI:** 10.1371/journal.pone.0110269

**Published:** 2014-10-22

**Authors:** Xinhua Yan, Sharath P. Sasi, Hannah Gee, JuYong Lee, Yongyao Yang, Raman Mehrzad, Jillian Onufrak, Jin Song, Heiko Enderling, Akhil Agarwal, Layla Rahimi, James Morgan, Paul F. Wilson, Joseph Carrozza, Kenneth Walsh, Raj Kishore, David A. Goukassian

**Affiliations:** 1 Cardiovascular Research Center, GeneSys Research Institute, Boston, Massachusetts, United States of America; 2 Calhoun Cardiology Center, University of Connecticut Health Center, Farmington, Connecticut, United States of America; 3 Tufts University School of Medicine, Boston, Massachusetts, United States of America; 4 Steward Carney Hospital, Dorchester, Massachusetts, United States of America; 5 Department of Integrated Mathematical Oncology, H. Lee Moffitt Cancer Center and Research Institute, Tampa, Florida, United States of America; 6 Biosciences Department, Brookhaven National Laboratory, Upton, New York, United States of America; 7 Steward St. Elizabeth's Medical Center, Boston, Massachusetts, United States of America; 8 Whitaker Cardiovascular Institute, Boston University School of Medicine, Boston, Massachusetts, United States of America; 9 Feinberg Cardiovascular Institute, Northwestern University, Chicago, Illinois, United States of America; Medical University Innsbruck, Austria

## Abstract

Previous epidemiologic data demonstrate that cardiovascular (CV) morbidity and mortality may occur decades after ionizing radiation exposure. With increased use of proton and carbon ion radiotherapy and concerns about space radiation exposures to astronauts on future long-duration exploration-type missions, the long-term effects and risks of low-dose charged particle irradiation on the CV system must be better appreciated. Here we report on the long-term effects of whole-body proton (^1^H; 0.5 Gy, 1 GeV) and iron ion (^56^Fe; 0.15 Gy, 1GeV/nucleon) irradiation with and without an acute myocardial ischemia (AMI) event in mice. We show that cardiac function of proton-irradiated mice initially improves at 1 month but declines by 10 months post-irradiation. In AMI-induced mice, prior proton irradiation improved cardiac function restoration and enhanced cardiac remodeling. This was associated with increased pro-survival gene expression in cardiac tissues. In contrast, cardiac function was significantly declined in ^56^Fe ion-irradiated mice at 1 and 3 months but recovered at 10 months. In addition, ^56^Fe ion-irradiation led to poorer cardiac function and more adverse remodeling in AMI-induced mice, and was associated with decreased angiogenesis and pro-survival factors in cardiac tissues at any time point examined up to 10 months. This is the first study reporting CV effects following low dose proton and iron ion irradiation during normal aging and post-AMI. Understanding the biological effects of charged particle radiation qualities on the CV system is necessary both for the mitigation of space exploration CV risks and for understanding of long-term CV effects following charged particle radiotherapy.

## Introduction

Previous epidemiologic data in radiotherapy patients [1]–[3], non-occupational exposure [4]–[6] and occupational exposure for ionizing radiation (IR)-induced cardiovascular (CV) diseases demonstrate that CV morbidity may occur within months or years, and CV mortality may occur within decades, after initial IR exposure. Most of what we know about harmful effects of IR on CV system is from terrestrial epidemiological studies of patients who are long-term survivors of conventional (X-ray or electron-based) [1]–[3] but not charged particle (protons, carbon ions) cancer radiotherapy (RT). The specific cause and effect relationship of IR effects on increased incidence of CV disease was reported in post-operative left- vs. right-sided RT, where left-sided RT was associated with 44% higher risk of death from CV disease compared to right-sided RT [1], [2]. In these procedures, equivalent single dose to the heart was determined to be 1–2 Gy, thus similar to the heart doses in the A-bomb survivors who developed fatal IR-induced CV disease decades after exposure [6]. A more recent epidemiologic study of 2,168 women who underwent conventional RT for breast cancer between 1958 and 2001 in Europe has shown that the rate of major coronary events increased linearly with the mean dose to the heart by 7.4% per Gy, *with no apparent lower or upper threshold* (overall average of the mean doses to the whole heart was 4.9 Gy, range −0.03 to 27.72 Gy) [7].

The effects of solar and galactic cosmic irradiation during and after space flights on the cardiovascular (CV) system are unknown. The majority of space flight-associated CV risks identified to date were determined shortly after low Earth orbit (LEO) International Space Station (ISS) flights, and include serious cardiac dysrhythmias, compromised orthostatic CV response, manifestation of previously asymptomatic CV disease and cardiac atrophy [8]–[10]. There is limited and sporadic epidemiologic data for long-term CV morbidity and mortality of US astronauts involved with the Mercury, Skylab, Shuttle and International Space Station (ISS) programs. In August 2013, the complete list of deceased US astronauts was released to public, including the cause of death, age and specific space program(s) participation. While limited, these data implicate heart attack as the second leading cause of death (after non-flight accidents) in astronauts who participated in the Apollo 11, 12, and 14–17 Moon missions. It appears that in later Shuttle and ISS programs, cancer is the second leading cause of death in US astronauts after flight and non-flight-related accidents. In order to better determine IR-induced CV risks associated with space travel beyond LEO, studies are performed at ground-based accelerator facilities which approximate the space radiation environment to determine proton and heavy ion effects in relevant in vitro and in vivo model systems. Such studies are essential to improve predictive risk models, as well as developing appropriate diagnostic tests and potential countermeasures.

During future Moon, near Earth asteroids or Mars missions, astronauts will be exposed to higher total doses (∼0.4–0.5 Gy) from galactic cosmic rays (GCR), especially during Mars missions that are currently estimated to be 22–32 months [9]. Measurements of the complex ionizing radiation environment inside the Curiosity rover while in transit to Mars determined that the dose equivalent for a short-duration roundtrip to be ∼0.66±0.12 Sv [10]. It is estimated that during deep space missions, each cell in an astronaut's body will be traversed by a proton (^1^H) every three days, a helium (^2^He) nucleus every few weeks, and high charge and energy (HZE) nuclei (e.g., ^12^C, ^16^O, ^28^Si, ^56^Fe, etc.) every few months [11]. In spite of the fact that only 1% of GCR is composed of ions heavier than helium, approximately 41% of IR dose-equivalent is predicted to be HZE particle-derived with 13% being from ^56^Fe particles alone. This is due to the extremely high linear energy transfer (LET; for iron tens to thousands of keV/µm in water versus ∼0.1–2 keV/µm for protons) and accompanying relative biological effectiveness (RBE) values of iron ions compared to protons [12]. The majority of astronauts are middle-aged (average age of 46), and thus at a higher risk for CV disease development [13]. During an exploration-class space mission to Mars, astronauts will not have access to comprehensive health care services for a period of at least 2 years [9], [14].

With essentially no information available on the effects of low dose charged particle IR on the CV system (i.e., heart), we examined the effects of acute, whole-body proton and iron ion IR on CV system alterations during normal aging and under induced ischemic conditions. In addition, we studied key molecular and cellular signaling pathways that may be responsible for these alterations. We believe that our findings not only benefit future NASA exploration-type space missions but also the general population, specifically hadron (i.e., proton and fast neutron) and heavy ion radiotherapy patients (e.g., carbon ions) [15], by providing initial mechanistic insights for development of CV morbidity and mortality due to charged particle irradiation.

To the best of our knowledge, no data are available on whether or not charged particle IR may pose significant additive risks for CV etiologies, or whether it impairs cardiac repair and regeneration processes following adverse CV events (*e.g.*, AMI). We report here, effects of charged particle IR typical of the space radiation environment on CV function up to 10 months after initial exposure in a mouse model with and without complication by an induced ischemic event. We also measured IR-induced changes in molecular and cellular pro-survival and angiogenesis signaling pathways related to CV function and recovery after AMI.

## Materials and Methods

### Animal models

Adult male C57Bl/6NT mice, 8–10 months of age (Taconic, Germantown, NY) were shipped directly to Brookhaven National Laboratory (BNL, Upton, NY). Mice were fed standard laboratory chow diet (Harlan Teklad), were given water access *ad libitum* and kept in the temperature- and light-controlled (12 hour light/dark cycles) environment. All mice were handled in accordance with the guidelines set and approved by the Institutional Animal Care and Use Committees (IACUC) at both GeneSys Research Institute (GRI) and Brookhaven National Labs (BNL). Any animal in this study found to exhibit severe or irreversible symptoms of pain and stress (limited mobility, reduced consumption of food and water, weight loss of 15% or more) was euthanized immediately by Pentobarbital based euthanasia solution 200 mg/Kg intraperitoneal (i.p). This method is consistent with the recommendation of the Panel on Euthanasia of the American Veterinary Medical Association *Guidelines on Euthanasia*.

### Particle Radiation and Dosimetry

Whole-body exposures of mice to 1 GeV protons (^1^H; LET = 0.223 keV/µm) and 1 GeV/nucleon (n) iron ions (^56^Fe; LET = 151.4 keV/µm) were performed at the NASA Space Radiation Laboratory at BNL according to standardized procedures. Animals were placed individually into rectangular polystyrene boxes with multiple air holes (4 mm in diameter) and then exposed to 50 cGy ^1^H or 15 cGy ^56^Fe ions in the Bragg plateau region. These doses were chosen to be approximately equitoxic for a variety of other relevant biological endpoints, assuming a RBE value of 1 for 1 GeV protons and ∼3 for 1 GeV/n ^56^Fe ions (compared to gamma ray reference radiation). The average dose-rate for the ^1^H and ^56^Fe irradiations was ∼16±5 cGy/min and 5±0.5 cGy/min respectively. Effective doses to the mouse heart were estimated using NASA GERMcode (the GCR Event-based Risk Model) Monte Carlo-based modeling algorithm. The GERMcode provides information of primary ion and fragment dose contributions as a function of distance into the target and provide charged particle dosimetry results in excellent agreement with physical NSRL Bragg curve measurements. One GeV/n ^56^Fe ion exposures, doses to the mouse heart (assumed to be ∼1 cm from the skin surface) were calculated to be ∼14.4 cGy with the LET reduced to ∼144 keV/micron (µm) at that position. For the 50 cGy 1 GeV proton exposures, the estimated heart dose would be ∼52.55 cGy and LET would be ∼0.217 keV/(µm). Note that GERMcode calculations reveal increased dose to the heart for protons and decreased dose to the heart for ^56^Fe ions (due to their differing fragmentation characteristics). Following irradiation, all animals were driven to GeneSys Research Institute in Boston from BNL for long-term housing and experimental analysis. Non-irradiated control mice underwent identical procedures including placement into polystyrene boxes and sham irradiation at NSRL.

### Short and Long-term Experimental Groups

We evaluated the effect of single low-dose whole body 50 cGy proton (^1^H) and 15 cGy, 1 GeV/n iron (^56^Fe) IR on the formation and disappearance of *p*-H2AX phospo-S139 foci (*p*-H2AX foci were evaluated up to 28 days post-IR, only), inflammation (CD68 staining) and oxidative DNA damage (8-oxo-de-oxy-Guanosine ELISA) in the hearts of 8–10 months old (at the time of initial IR) C57BL/6NT mice at various times up to 3 months post-IR. Each group (^1^H, ^56^Fe and N-IR) consisted of *n* = 8 animals per time point for short-term studies: 2 h, 5 h and 24 h and long-term studies: 7 days, 14 days, 28 days, 2 months and 3 months.

### Immunofluorescent Staining, Imaging and Analysis

#### DNA damage and repair

To assess the formation and decay of *p*-H2AX foci 6–8 µm section of OCT embedded heart tissue from sham-irradiated, proton and ^56^Fe-ion-irradiated mice collected at various times up to 28 days post-IR, were fixed in 4% paraformaldehyde (PFA) for 15 min at room temperature (RT) and washed with 1× phosphate buffered saline (1×PBS) for 5 min. Sections were permeabilized with 0.1% Triton X-100 (Sigma, St. Louis, MO) for 15 min at RT and washed three times in 1×PBS for 5 min. Primary anti-*p*-H2AX rabbit monoclonal antibody (Cell Signaling Technology, Danvers, MA) and biotinylated isolectin-B4 (ISB4 an EC-marker, Life Technologies, Grand Island, NY) followed by Alexa-488 goat anti-rabbit secondary antibody (Life Technologies) and Alexa-594 labeled streptavidin (Life Technologies) were used respectively to assay *p*-H2AX foci formation and decay over time in cardiac endothelial cells-ECs (i.e., double positive for ISB4 and *p*-H2AX) and cardiac non-EC cells (*p*-H2AX positive and ISB4 negative). Cardiac non-ECs may represent cardiomyocytes (CM), cardiac fibroblasts and inflammatory cells. Topro-3 was used to visualize nuclei (Life Technologies). Images were obtained using laser scanning confocal microscope (LSM 510 Meta, ZEISS, Thornwood, NY) at 1000× magnification. Cells with apoptotic features or micronuclei were not considered for *p*-H2AX analysis. Data were obtained from three replicate samples for sham-irradiated, proton and ^56^Fe ion-irradiated cardiac tissues. Using a computer-assisted image analysis algorithm based on pixel and color distribution the *p*-H2AX foci were evaluated by quantifying all cells with ≥1 *p*-H2AX foci. Graphs were plotted for mean foci/cell and for a percent of cells with an N of *p*-H2AX foci.

#### Inflammation/Macrophages

To evaluate inflammatory infiltration the expression of CD68, a glycoprotein normally expressed on macrophages, also known in mice as macrosialin was quantified. Heart tissue sections from all three groups were processed similarly as detailed above and stained with rat anti-mouse CD68 monoclonal antibody (AbD Serotec, Raleigh, NC) along with Alexa-488 goat anti-rat secondary antibody (Life Technologies) and Topro-3. Images were obtained using laser scanning confocal microscope at ×200 magnification.

### Enzyme-Linked Immunosorbent Assay (ELISA) for Oxidative Damage

DNA extracts were obtained from sham-irradiated, proton and ^56^Fe-ion-irradiated mice heart tissue collected at various time points up to 3 months post-IR using DNA isolation kit as per manufacturer protocol (Qiagen, Valencia, CA). Isolated DNA was converted to single-stranded DNA and denatured to be processed for 8-Oxo-de-oxy-Guanosine ELISA (Cell Biolabs Inc., San Diego, CA) according to detailed manufacturer protocols (*n* = 3/group/time point). The plates were read using Tecan Spectra model 96 Well Microplate Reader (MTX Lab Systems, Vienna, VA) using 450 nm primary wavelength.

### Radiation+Aging (IR+Aging) Group

To evaluate the effects of a single low-dose full body IR in the heart, we exposed 8–10 months old C57BL/6NT to ^1^H and ^56^Fe IR and IR-induced alterations in cardiac function, tissue, cellular and molecular changes in IR+Aging groups post-IR at 1, 3 and 10 months, were assessed by ECHO and HEMO, cardiac fibrosis by Masson's Trichrome and H&E staining and activation of signaling pathways by protein analyses.

### Radiation + Aging + AMI Group and Acute Myocardial Infarct Surgery

We also evaluated the effect of low-dose whole-body acute ^1^H and ^56^Fe ion irradiation in the hearts of 8–10 months old C57BL/6NT over 10 months in an AMI model. AMI was induced by ligation of LAD coronary artery 1, 3 and 10 months post-IR as described before [16], [17] and mice were monitored over 28 days post-AMI by ECHO and before sacrificing HEMO measurements were performed on day 28.

### Cardiac Physiology

#### Echocardiography (ECHO)

After measurement of body weight, animals were lightly anesthetized with isoflurane vaporized in O_2_ (1.5–2%) at the rate of 1 L/min using a nose cone on a warming pad. 2D guided M-mode ECHO was performed with a 15-MHz (15–6L) pediatric open heart surgery transducer (Agilent Technologies, Santa Clara, CA) as described before [18]. At least 5 sequential beats were analyzed (*n* = 6–8/group). Heart rate, LV end diastolic diameter (EDD) and end systolic diameter (ESD) and LV wall thickness were measured. LV ejection fraction (EF%) and fractional shortening percent (FS%) were calculated using standard formulas EF% = (EDV−ESV)/EDV×100 and FS% = (EDD−EDS)/EDD×100.

#### Hemodynamic (HEMO)

In vivo LV pressure measurements were performed by direct LV catheterization using Millar Mikro-Tip Blood Pressure System (1.2F, SciSense, Ithaca, NY) (*n* = 6–8/group) as described previously [18]. Heart rate, LV systolic pressure (LVSP), LV end-diastolic pressure (LVEDP), dP/dt_max_ and dP/dt_min_ were recorded.

### Histology, Imaging and Analysis

#### Routine Histology, H&E staining

Sections (10 µm) of formalin fixed paraffin embedded hearth tissue were stained with H&E and visualized using a light microscope (Leica Microsystems, Buffalo Grove, IL). Images of the full circumference of the heart cross sections along with the infarct area post-AMI were taken at ×100 magnification and collaged.

#### IR-Induced Fibrosis, Masson's Trichrome Staining

To determine whether low dose ^1^H- or ^56^Fe-IR could induce cardiac fibrosis 1, 3 and 10 months post-IR, serial 10 µm sections of cardiac tissue were processed for Masson's Trichrome staining (Electron Microscopy Sciences, Hatfield, PA) and random regions of the heart were imaged at ×200 (*n* = 20 images/sample per group) to be analyzed using Image-J program (v1.40, NIH) to quantify for percentage of fibrosis (blue pixels). Also full circumference bright-field microscope images (×100) of at least 40–50 cross sections of the right and left ventricles along with infarct region of hearts at day 28 post-AMI were analyzed using Image-J to quantify infarct size as described [19].

#### Initial Infarct Size

To evaluate and compare the initial infarct size in sham-irradiated, proton and ^56^Fe ion-irradiated mice we collected heart samples 3 days post-AMI for 1, 3 and 10 month time points. Heart samples were processed as described earlier and 6–8 µm section of OCT embedded heart tissue were double immunostained with primary cTnI antibody (Santa Cruz Biotechnology Inc. Santa Cruz, CA) followed with Alexa-555 goat anti-rabbit secondary antibody (Life Technologies) and Topro3. Confocal images of stained tissue were taken at ×100 magnification and analyzed using Image-J software.

### Western Blot Analysis

To evaluate the various signaling pathways that regulate cardiac functions post-IR and post-AMI at 1, 3 and 10 month time points we performed Western blot analysis at different time points. LV tissue (*n* = 3–5/treatment group) at different times after IR (1, 3 and 10 months) and post-AMI (3, 7, 14 and 28 days) were snap-frozen in liquid nitrogen immediately after collection. LV tissue was homogenized using tissue homogenizer in a buffer containing 20 mM Tris (pH 7.5), 150 mM NaCl, 1 mM EDTA, 1 mM EGTA, 1% Triton X-100, 10 mM sodium pyrophosphate, 20 mM β-glycerophosphate, 10 mM Na_3_VO_4_, 1 mM NaF, 1 mM PMSF, and protease inhibitor cocktail tablet (Roche Diagnostics, Indianapolis, IN). Samples containing 50 µg of total protein from the LV tissue lysates were mixed with equal volume of ×2 sample buffer and boiled for 5 min at 95°C. Protein fractions were then separated by electrophoresis on a 8%, 10% or 15% polyacrylamide gel (Bio-Rad, Waltham, MA) and blotted onto PVDF membrane. Detection of total (T) and phosphorylated (p) protein levels of Akt, Erk1/2, cTnI, p38 and S6k were performed using antibodies against phospho-specific or total-specific antibodies (Cell Signaling Technology Inc.).

For NCX and SERCA2a proteins we used monoclonal antibodies (Thermo Scientific, Tewksbury, MA) at 1∶1000 dilutions along with HRP-linked horse anti-mouse IgG secondary antibody at 1∶1000 dilution (Cell Signaling Technology Inc.). Detection of total (T) and phosphorylated (p) protein levels of NFATc4 was performed using rabbit polyclonal antibodies (Santa Cruz Biotechnology Inc.) and HRP-linked goat anti-rabbit secondary. GAPDH (1∶40,000 dilution, Millipore, Billerica, MA) expression was used to adjust the protein loading. Protein levels were revealed using enhanced chemiluminescence western blotting method (ECL, Fisher Scientific). Densitometry analysis of the western blot band brightness was used to obtain total and phosphorylated levels of a protein. Image J software was used to measure the band intensities for each respective sequence of phosphorylated and total protein, and corresponding loading control.

### Statistical Analysis

Results in all graphs are expressed as mean ± SEM. Statistical analyses were performed by using one-way ANOVA (Stat View Software, SAS Institute Inc., Middleton, MA). Differences were considered significant at *P*<0.05.

## Results

### DNA Damage Responses after ^1^H- and ^56^Fe-IR in the Heart Tissue

#### Kinetics of IR-induced p-H2AX foci

**Figure 1:**
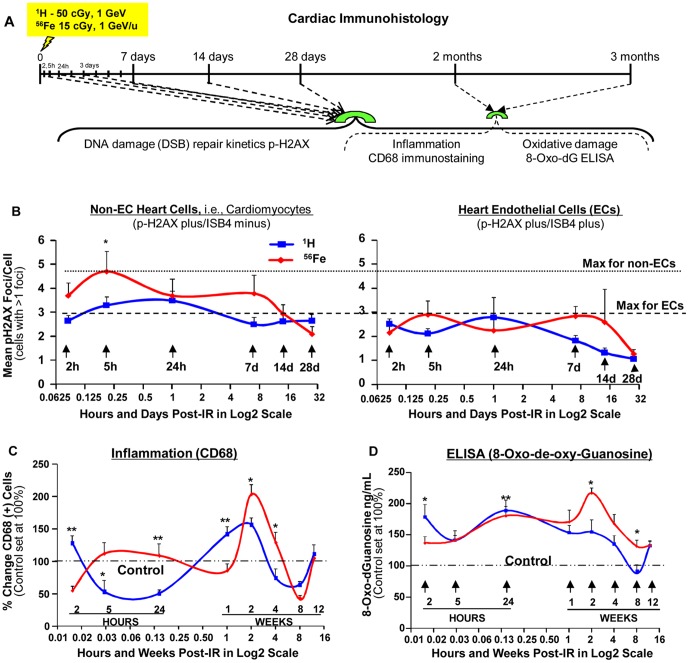
Formation and the Decay of *p*-H2AX Foci, Inflammatory and Oxidative DNA damage Responses. (**A**) Diagrammatic representation of the experimental design to evaluate the effect of a single low-dose full body exposure to 50 cGy, 1 GeV ^1^H and 15 cGy, 1 GeV/n ^56^Fe ions on the formation and disappearance of *p*-H2AX foci, inflammation (CD68 staining), and oxidative DNA damage (8-oxo-deoxy-Guanosine ELISA) in the hearts of 8–10 months old (at the time of initial IR) C57BL/6NT mice over 28 days post-IR. CD68 staining and 8-oxo-dG ELISA were also performed at 2 and 3 months; (**B**) Graphic representation of mean *p*-H2AX foci/cell (cells with >1 foci) in non-EC (*p*-H2AX plus/ISB4 minus) cells and ECs (*p*-H2AX plus/ISB4 plus) in the heart tissue isolated from mice of sham-irradiated, ^1^H-IR and ^56^Fe-IR up to 28 days. In figures **B** and C the time points on the X-axis are represented in Log^2^ scale. Dotted line represents the maximum *p*-H2AX foci/cell for non-ECs and dashed line represents maximum *p*-H2AX for ECs. *P<0.05 for ^1^H vs. ^56^Fe. (**C**) Graphic representation of % change in CD68 positive (+) cells in the heart tissue of non-IR, ^1^H-IR and ^56^Fe-IR mice within hours and up to 12 weeks post-IR. CD68 (+) cells in control hearts was set at 100% (dash-dotted line). *P<0.01 and **P<0.001 for ^1^H vs. ^56^Fe. (**D**) Graphic representation of percent change in IR-induced 8-oxo-dG levels, quantified by ELISA in heart tissue isolated from sham-irradiated, ^1^H-IR and ^56^Fe-IR mice up to 12 weeks. The level of 8-oxo-dG in control hearts was set at 100% (dash-dotted line). *P<0.005 for ^1^H vs. ^56^Fe All results are presented as mean ± SEM; *n* = 6–8 animals per time point/group for sham controls, ^1^H-IR (solid blue line) and ^56^Fe-IR (solid red line) groups. Statistical significance was assigned when *P*<0.05.

We studied the kinetics of *p-*H2AX foci, a well-characterized marker of DNA double strand breaks (DSB) [20], by measuring foci levels in cardiac tissues over the course of 28 days post-irradiation. Following either 50 cGy of 1 GeV protons or 15 cGy of 1 GeV/n ^56^Fe ions, IR-induced mean *p-*H2AX foci levels (corrected for spontaneous background levels) were higher in non-endothelial cells (non-EC; represented primarily by cardiomyocytes) versus endothelial cells (EC) in the heart tissue. Compared to non-IR control heart tissue (1.5–1.75 *p-*H2AX foci/cell), ∼2–5-fold increases in *p-*H2AX foci levels were observed 2–24 hours post-IR in both EC and non-EC cells and remained significantly increased above background levels for 7 days (**Fig. 1B** **and Fig. S1A–B**). Further, the disappearance of ^56^Fe ion-induced *p-*H2AX foci was significantly slow, with residual *p-*H2AX foci observed 28 days post-IR in non-EC cells.

#### Inflammatory Infiltration

We found that proton or ^56^Fe ion-induced inflammatory responses showed cyclical response patterns up to 3 months post-IR (**Fig. 1C** **and Fig. S2A–B**). Persistent inflammatory responses may lead to release of various cytokines, superoxide, nitric oxide and other signaling molecules by immune cells (i.e., macrophages), which are capable of causing oxidative tissue damage [21].

#### Oxidative DNA damage – 8-Oxo-de-oxy-Guanosine (8-oxo-dG) ELISA

We measured the level of oxidative DNA damage by 8-Oxo-de-oxy-Guanosine (8-oxo-dG) ELISA. In proton-irradiated cardiac tissues, increased levels of 8-oxo-dG were detected at 2 and 24 hours post-IR. In ^56^Fe ion-irradiated cardiac tissues, significant increases were not observed until 24 hours with the maximum 8-oxo-dG levels detected at 2 weeks (**Fig. 1D**), suggesting that ^56^Fe ion irradiation induced longer-lasting oxidative damage responses in the heart.

### Longitudinal Studies, Radiation and Aging Model

The survival among control, proton-irradiated and ^56^Fe ion-irradiated mice up to 10 months was 95–98% (*n* = 100 animals/treatment group), and no statistical differences were observed between control and either IR groups up to 10 months. IR-induced alterations in cardiac functions were assessed by echocardiography (ECHO) and hemodynamic (HEMO) measurements; activation of signaling pathways was assessed by Western blot analyses.

#### Cardiac Physiology and Fibrosis

**Figure 2:**
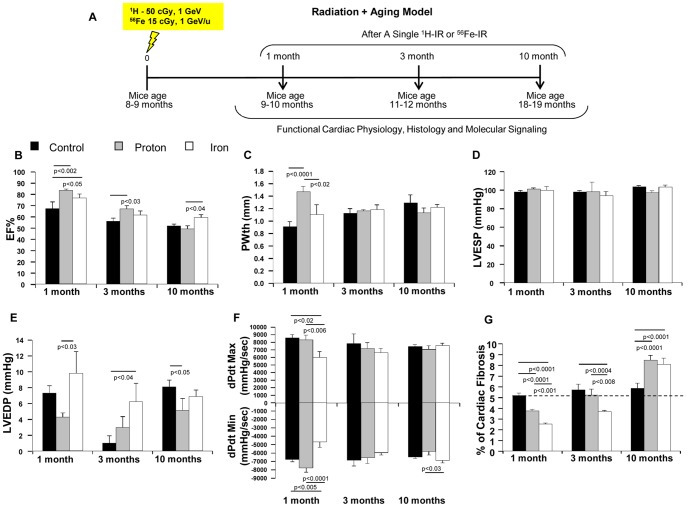
ECHO and HEMO Measurements of Cardiac Functions and Evaluation of Cardiac Fibrosis in IR + Aging Model. (**A**) Diagrammatic representation of the experimental design to evaluate the effect of acute low-dose, whole body 50 cGy, 1 GeV ^1^H and 15 cGy, 1 GeV/n ^56^Fe IR in the hearts of 8–10 months old C57BL/6NT over 10 months post-IR. IR-induced alterations in **Radiation+Aging** model in cardiac function were assessed by echocardiography (ECHO), hemodynamic (HEMO) and morphometric/histologic measurements and activation of signaling pathways by protein analyses. *ECHO analysis of cardiac function in the hearts of full-body ^1^H-IR, ^56^Fe-IR and non-IR control mice 1, 3 and 10 months post-IR for:* (**B**) Ejection Fraction - EF%, (**C**) Posterior wall thickness - PWth (mm). *HEMO measurements and analysis of cardiac function in the hearts of full-body ^1^H-IR, ^56^Fe-IR and non-IR control mice 1, 3 and 10 months post-IR for:* (**D**) LV ESP (mmHg), (**E**) LV EDP (mmHg), (**F**) LV dP/dt_Max_ and dP/dt_Min_ (mmHg/sec). (**G**) Graphic representation of % fibrosis in the hearts of full-body ^1^H-IR, ^56^Fe-IR and non-IR control mice 1, 3 and 10 months post-IR evaluated with Masson's trichrome staining. Results in all graphs (**B–G**) are presented as mean ± SEM; *n* = 6–8 animals per time point/group for non-IR control (solid black bars), ^1^H-IR (solid grey bars) and ^56^Fe-IR (solid white bars). Statistical significance was assigned when *P*<0.05.

Hemodynamic and echocardiography studies showed that in proton-irradiated hearts, left ventricular (LV) posterior wall thickness (PWth) was increased at 1 month (**Fig. 2C**), which was associated with lower LV end-diastolic pressure (LVEDP) (**Fig. 2E**), as well as increased ejection fraction % (EF%) (**Fig. 2B**) and dP/dt_min_ compared to controls (**Fig. 2F**), indicating the development of cardiac hypertrophy. LV PWth was decreased during 3–10 months after proton irradiation (**Fig. 2C**), which was associated with the decrease of LV dP/dt_max_ and dP/dt_min_ (**Fig. 2F**) and increase of cardiac fibrosis compared to the hearts at 1 month (**Fig. 2G** **and Fig. S3D–I**). In control mice, LV PWth gradually increased with comparable dP/dt_max_ and dP/dt_min_ (**Fig. 2C and 2F**), whereas cardiac fibrosis remained unaffected during at least the first 3 months (**Fig. 2G**). These results suggest that proton-irradiated hearts may transit from cardiac hypertrophy to failure 3–10 months post-IR. We also observed that LVEDP was increased 1 and 3 months after ^56^Fe ion irradiation (**Fig. 2E**). In addition, dP/dt_max_ and dP/dt_min_ were decreased at 1 month (**Fig. 2F**), suggesting that ^56^Fe ion-irradiated hearts developed systolic and diastolic dysfunction as early as 1 month post-IR. This, however, was not associated with increased cardiac fibrosis at 1 and 3 months (**Fig. 2G**). In ^56^Fe ion-irradiated hearts cardiac function returned to control levels at 10 months (**Fig. 2E and 2F**).

#### Candidate Pathways/Molecular Mechanisms

**Figure 3:**
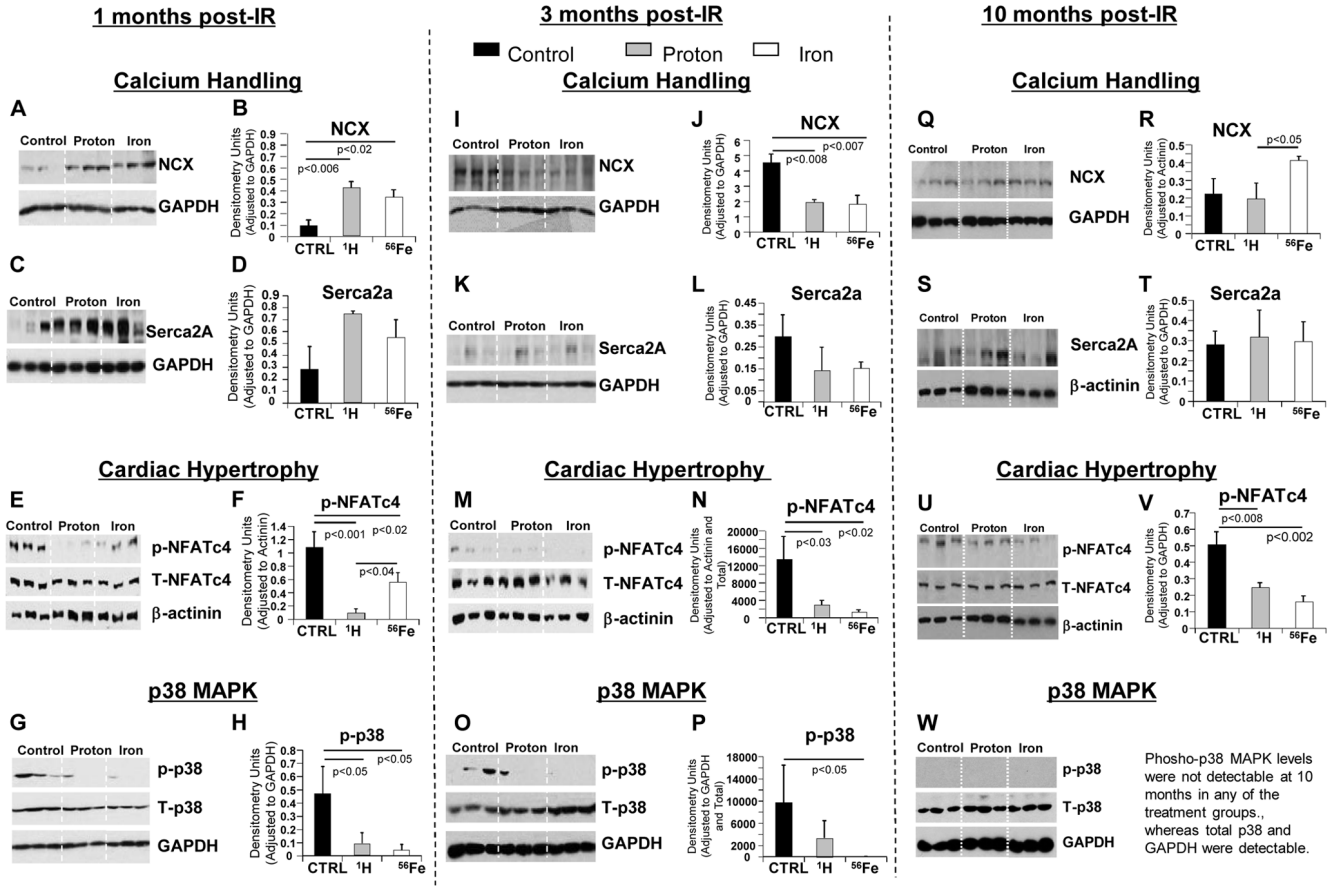
IR-induced Changes in Signaling Pathways 1, 3 and 10 months post-IR. Representative western blot scans of heart tissue homogenates from sham controls, ^1^H- and ^56^Fe-IR mice at 1, 3 and 10 months post-IR in **IR+Aging model**. Bands represent phosphorylated (p), total (T) and loading control for the following proteins - NCX and GAPDH (**A, I, Q**), SERCA2a and GAPDH (**C, K, S**), p-NFATc4, T-NFATc4 and β-actinin (**E, M, U**), p-p38, T-p38 and GAPDH (**G, O, W**). Quantification and graphic representation of total protein levels and phosphorylation using densitometric analysis of phospho-band intensities after adjusting for corresponding GAPDH/β-actinin and total band intensities of heart tissue homogenates at 1, 3 and 10 months post-IR for the following proteins - NCX (**B, J, R**), SERCA2a (**D, L, T**), p-NFATc4 (**F, N, V**), p-p38 (**H and P**). Results in all graphs represent mean ± SEM of the pooled data from *n* = 6–8 animals per time point/group for non-IR control (solid black bars), ^1^H-IR (solid grey bars) and ^56^Fe-IR (solid white bars) groups. Statistical significance was assigned when *P*<0.05.

To determine underlying molecular mechanisms that may be responsible for the changes in cardiac function during 10 months post-IR, we evaluated the expression and the activity of proteins that are involved in the regulation of Ca^2+^ handling, p38 MAPK activity, cardiomyocyte (CM) contractility and hypertrophy in control, proton and ^56^Fe ion-irradiated cardiac tissues. At 1 month post-IR, sodium calcium exchanger (NCX) and Sarcoplasmic Reticulum Ca^2+^ ATPase (SERCA2a), proteins critical for proper calcium handling [22], were significantly up-regulated (**Fig. 3A–D**) followed by a decrease of both proteins at 3 months after proton or ^56^Fe ion irradiation indicating that exposure to both types of charged particles altered proper cardiac calcium homeostasis (**Fig. 3I–L**). SERCA2a levels returned to un-irradiated control levels by 10 months in proton and ^56^Fe ion-irradiated hearts, whereas NCX levels were again increased after ^56^Fe ion irradiation (**Fig. 3Q–T**).

Phosphorylated NFATc4 (Nuclear Factor of Activated T cells, cytoplasmic, calcineurin-dependent 4) was used to assess activation and onset of cardiac hypertrophy. Compared to control hearts, we observed a decrease of NFATc4 phosphorylation in both proton and ^56^Fe ion-irradiated hearts 1, 3 and 10 months post-IR (**Fig. 3E, F, M, N and U, V**, respectively). Decreased phosphorylation of NFATc4 indicates increased activity, suggesting activation of cardiac hypertrophy signaling in both proton and ^56^Fe ion-irradiated hearts over 10 months. Decreased phosphorylation of NFATc4 was associated with a decrease of p38 MAPK phosphorylation, a kinase that regulates NFATc4 phosphorylation [23], in both proton and ^56^Fe ion-irradiated hearts 1, 3 and 10 months post-IR (**Fig. 3G, H, O, P and W**, respectively).

### Longitudinal Studies, Radiation and Aging in the Setting of Ischemia - AMI

Left anterior descending (LAD) coronary artery ligation was performed 1, 3 and 10 months after proton and iron irradiation. For all groups combined, AMI surgery mortality that occurred within 1–2 days post-LAD ligation was 14±1.5%, 11±6% and 10±3.2% at 1, 3 and 10 months post IR, respectively (N = 24–32 mice/treatment group). Post-AMI survival after LAD ligation was not significantly different among non-IR control, proton, and ^56^Fe ion-irradiated groups up to 28 days post-AMI (100%, 100% and 88±13% survived AMI surgery, respectively, p = ns). To assure that initial infarct size was similar for all IR groups, we evaluated three mice per treatment group. Three days post-AMI, hearts were isolated, fixed and stained for cardiac troponin I (cTnI), and showed no differences among non-IR+AMI, ^1^H-IR+AMI and ^56^Fe-IR+AMI groups (**Fig. S4A–B**).

#### Cardiac Physiology

**Figure 4:**
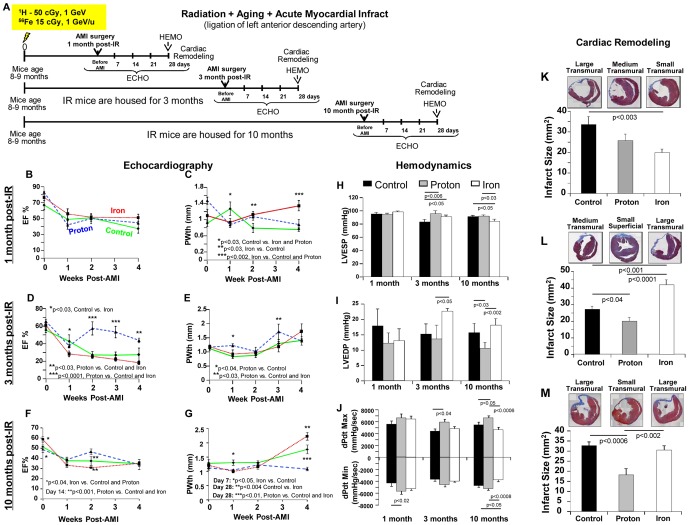
ECHO, HEMO Measurements of Cardiac Functions and Cardiac Remodeling in IR + Aging + AMI Model. (**A**) Diagrammatic representation of the experimental design to evaluate the effect of acute, low-dose, whole body 50 cGy 1 GeV ^1^H and 15 cGy 1 GeV/n ^56^Fe IR in the hearts of 8–10 months old C57BL/6NT over 10 months in **Radiation + Aging + AMI**. IR-induced alterations in cardiac function were assessed by echocardiography (ECHO), hemodynamic (HEMO) and morphometric/histologic measurements and activation of signaling pathways by protein analyses. Acute myocardial infarct (AMI) was induced by ligation of the left anterior descending (LAD) coronary artery 1, 3 and 10 months post-IR, and mice were monitored over 28 days post-AMI. *ECHO analysis of cardiac function in the hearts of full-body ^1^H-IR, ^56^Fe-IR and non-IR control mice 1, 3 and 10 months post-IR in IR+Aging+AMI model for:* EF% 1 month (**B**), 3 months (**D**), 10 months (**F**), PWth (mm) 1 month (**C**), 3 months (**E**) and 10 months (**G**). Results in all graphs (**B–G**) are presented as mean ± SEM; *n* = 6–8 animals per time point/group. Non-IR control – solid green line, ^1^H-IR - dashed blue line and ^56^Fe-IR - dotted red line. *HEMO measurements and analysis of cardiac function in the hearts of full-body ^1^H-IR, ^56^Fe-IR and non-IR control mice 1, 3 and 10 months post-IR for:* (**H**) LV ESP (mmHg), (**I**) LV EDP (mmHg), (**J**) LV dP/dt_Max_ and dP/dt_Min_ (mmHg/sec). Results in all graphs (**H–J**) are presented as mean ± SEM; *n* = 6–8 animals per time point/group for non-IR control - solid black bars, ^1^H-IR - solid grey bars and ^56^Fe-IR - solid white bars. Statistical significance was assigned when *P*<0.05. *Cardiac Remodeling 1, 3 and 10 months post-IR and 28 days after AMI:* Cardiac fibrosis was measured in the heart tissue post-AMI using Masson's Trichrome staining - blue is fibrosis and dotted line indicates the infarct scar size. Measurements represent midline length of the infarct when >50% of the LV was involved (mm) and every 3^rd^ section of the adjacent 8 µm size section were measured and infarct size was reconstructed as described before. Insets are representative images of ^1^H-IR, ^56^Fe-IR and non-IR control mice 1 month (**K**), 3 months (**L**) and 10 months (**M**) post-IR and 28 days after AMI. Graphic representation of the infarct size/scar (mm^2^) 1 month (**K**), 3 months (**L**) and 10 months (**M**) post-IR and 28 days after AMI. Results in all graphs are presented as mean ± SEM; *N* = 6–8 animals per time point/group for non-IR control - solid black bars, ^1^H-IR - solid grey bars and ^56^Fe-IR - solid white bars. Statistical significance was assigned when *P*<0.05.

We observed that proton-irradiated mice had improved cardiac function compared to control mice when AMI was induced 3 and 10 months post-IR (**Fig. 4D–J, L–M**). Compared to non-IR+AMI mice, ^1^H-IR+AMI mice had higher dP/dt_max_ and dP/dt_min_ and smaller infarct size (**Fig. 4J, L, M** **and Figs. S5B–C, S7A–B and S8A–B**). In addition, 3 and 10 month post-IR and 28 days after AMI, EF% and PWth in ^1^H-IR+AMI hearts were comparable to those before AMI (**Fig. 4D–E and F–G**). It is conceivable that low-dose proton irradiation may have some type of preconditioning effect that is reminiscent of ischemic pre-conditioning in the heart [24].

The results with the AMI model further highlight the inability of the heart to recover after 15 cGy, 1 GeV/n ^56^Fe ions, especially when AMI was induced later at 3 and 10 months post-IR (**Fig. 4D–J and L–M**). When AMI was induced 3 months after IR, LVEDP was significantly increased, and the infarct size was larger in ^56^Fe-IR+AMI hearts (**Fig. 4I, L** **and Figs. S5B, S7C**). When AMI was induced 10 months after IR, dP/dt_max_ and dP/dt_min_ were lower (**Fig. 4J**), the LV wall was thicker (**Fig. 4G**) and infarct size was larger in ^56^Fe-IR+AMI hearts 28 days after AMI (**Fig. 4M** **, and Figs. S5C, S8C**). Compared to control, both ^1^H- and ^56^Fe-IR improved cardiac remodeling at 1 month (post-AMI scar formation) (**Fig. 4K** **and Figs. S5A, S6A–C**).

#### Candidate Pathways/Molecular Mechanisms

**Figure 5:**
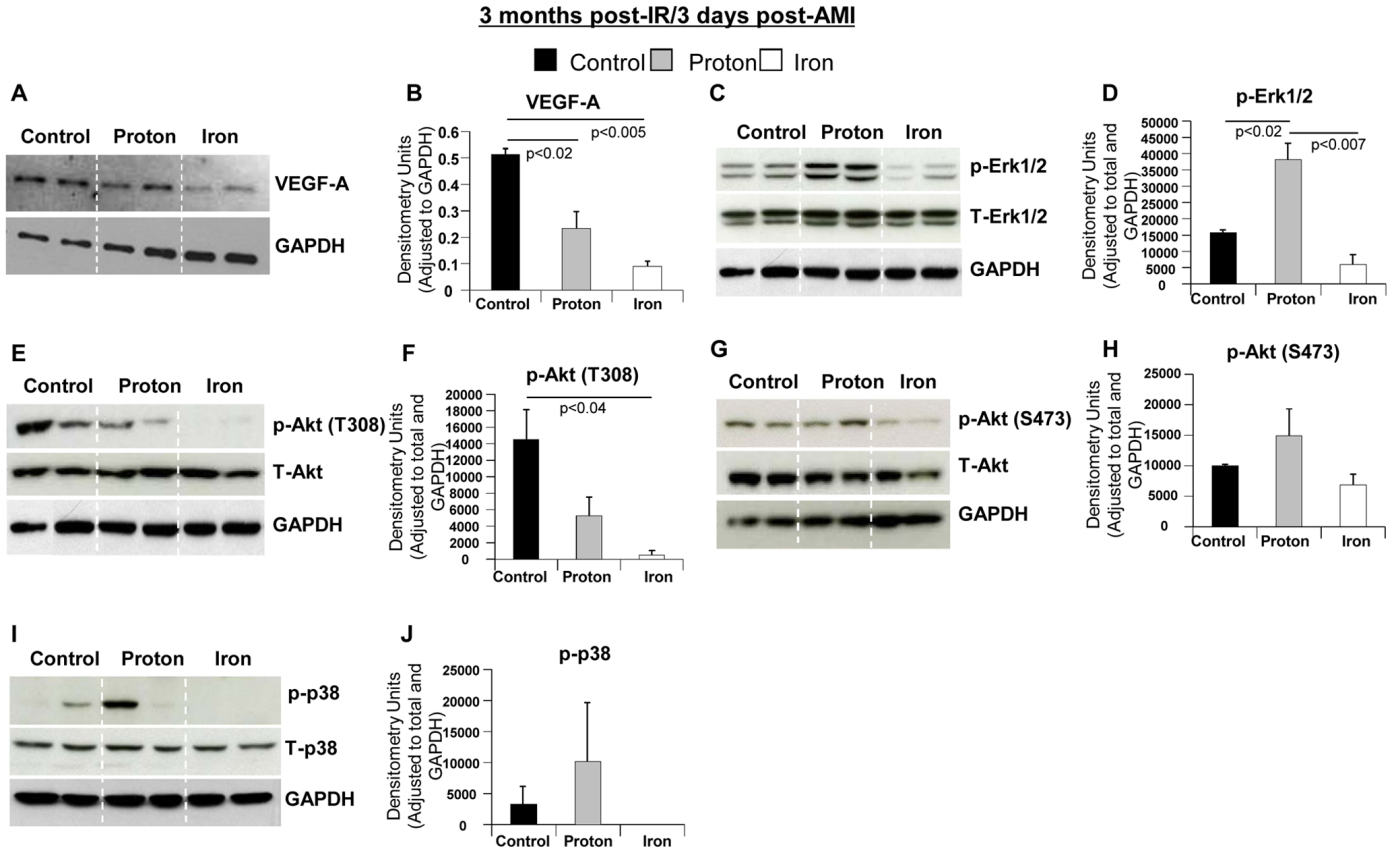
Molecular Pathways 3 days post-AMI in ^1^H-IR, ^56^Fe-IR and sham control hearts at 3 months post-IR. Representative Western blot images of heart tissue homogenates from sham control, ^1^H- and ^56^Fe-IR mice at 3 months post-IR in **IR+Aging+AMI model**. Images represent phosphorylated (p), total (T) and loading control for the following proteins (**A**) VEGF-A and GAPDH, (**C**) p-Erk1/2, T-Erk1/2 and GAPDH, (**E**) p-Akt (T308), T-Akt and GAPDH, (**G**) p-Akt (S473), T-Akt and GAPDH, (**I**) p-p38, T-p38 and GAPDH. Quantification and graphic representation of total protein levels and phosphorylation using densitometric analysis of phospho-band intensities after adjusting for corresponding GAPDH/β-actinin and total band intensities of heart tissue homogenates at 3 months post-IR and 3 days after AMI for the following proteins - (**B**) VEGF-A, (**D**) p-Erk1/2, (**F**) p-Akt (T308), (**H**) p-Akt (S473), (**J**) p-p38. Results in all graphs are depicted as mean ± SEM values and represent a pooled data from *n* = 6–8 animals per time point/group for non-IR control (solid black bars), ^1^H-IR (solid grey bars) and ^56^Fe-IR (solid white bars) groups. Statistical significance was assigned when *P*<0.05.

To determine the underlying molecular mechanisms associated with long-lasting effects of proton or ^56^Fe ion irradiation on post-ischemic recovery, we analyzed key signaling molecules that regulate angiogenesis, survival and proliferation, including VEGF-A, Akt, Erk1/2 and p38 [25]–[27]. We observed that in ^1^H-IR+AMI hearts, the level of VEGF-A and the phosphorylation of Akt, Erk1/2 and p38 were maintained or less depressed compared to non-IR+AMI hearts. The phosphorylation of Erk1/2 was even higher in ^1^H-IR+AMI vs. non-IR+AMI hearts (**Fig. 5C–D**). In contrast, phosphorylation of these signaling molecules were significantly reduced in ^56^Fe-IR+AMI hearts 3 days after AMI (3 months post-IR) (**Fig. 5A–J**). Decreases of angiogenic and pro-survival signaling molecules (**Fig. 5A–J**) may partially be responsible for the worsened cardiac function observed in ^56^Fe-IR+AMI hearts [28].

**Figure 6:**
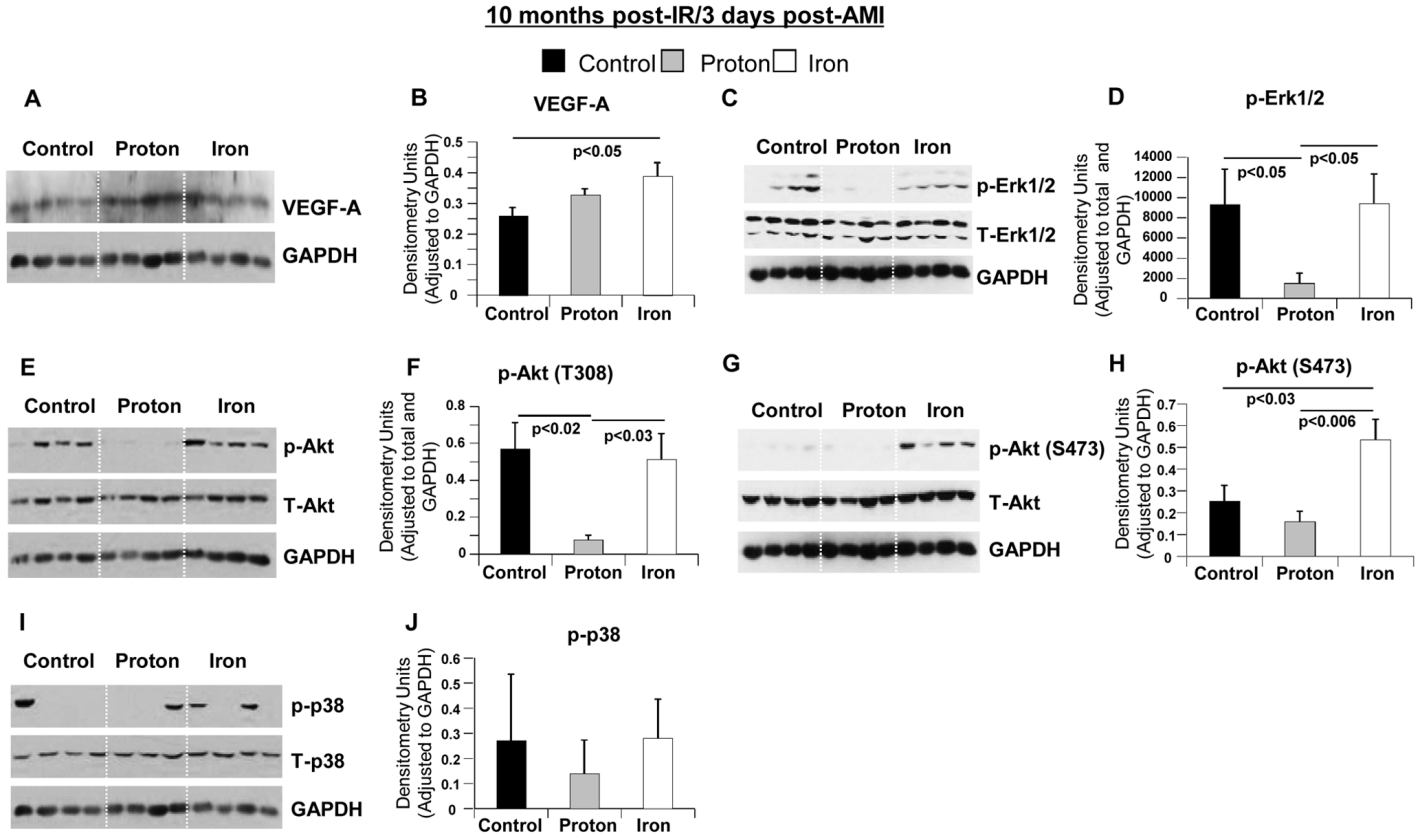
Molecular Pathways 3 days post-AMI in ^1^H-IR, ^56^Fe-IR and non-IR control hearts at 10 months post-IR. Representative Western blot images of heart tissue homogenates from sham control, ^1^H- and ^56^Fe-IR mice at 10 months post-IR in **IR + Aging + AMI model**. Images represent phosphorylated (p), total (T) and loading control for the following proteins (**A**) VEGF-A and GAPDH, (**C**) p-Erk1/2, T-Erk1/2 and GAPDH, (**E**) p-Akt (T308), T-Akt and GAPDH, (**G**) p-Akt (S473), T-Akt and GAPDH, (**I**) p-p38, T-p38 and GAPDH. Quantification and graphic representation of total protein levels and phosphorylation using densitometric analysis of phospho-band intensities after adjusting for corresponding GAPDH/β-actinin and total band intensities of heart tissue homogenates at 10 months post-IR and 3 days after AMI for the following proteins - (**B**) VEGF-A, (**D**) p-Erk1/2, (**F**) p-Akt (T308), (**H**) p-Akt (S473), (**J**) **p**-p38. Results in all graphs are depicted as mean ± SEM values and represent a pooled data from *n* = 6–8 animals per time point/group for non-IR control (solid black bars), ^1^H-IR (solid grey bars) and ^56^Fe-IR (solid white bars) groups. Statistical significance was assigned when *P*<0.05.

However, 3 days after AMI in 10 months post-IR hearts, we observed increased levels of VEGF-A, Erk1/2 and Akt in ^56^Fe-IR+AMI hearts compared to ^1^H-IR+AMI hearts (**Fig. 6A–H**). No difference was observed in p38 MAPK activation among all treatment groups post-AMI at 10 months (**Fig. 6I–J**). These increased levels which were comparable to non-IR+AMI hearts, may indicate a potential need for increased angiogenesis, survival and growth signaling in ^56^Fe-IR+AMI hearts that suggest a greater demand for cell survival signaling perhaps due to more severe ischemia-induced cardiac damage in ^56^Fe ion-irradiated hearts.

## Discussion

DNA damage responses following a single low-dose (≤0.1 to 0.5 Gy) proton or ^56^Fe ion irradiation in whole body-irradiated mouse cardiac tissues have not been evaluated before. Our study revealed that over 28 days proton or ^56^Fe ion-induced *p-*H2AX foci, a well-characterized marker for the formation and disappearance of DNA DSBs [20], were higher in non-endothelial cells (non-EC), represented primarily by cardiomyocytes (CM), versus heart endothelial cells (EC). Further, the decay of proton or ^56^Fe ion-induced *p-*H2AX foci in cardiac tissue was significantly slow with residual *p-*H2AX foci observed 28 days post-IR in non-ECs. We found that proton or ^56^Fe ion-induced inflammatory responses have cyclical patterns up to 3 months post-IR, with ^56^Fe ion irradiation inducing longer-lasting oxidative damage responses. This may provide a potential feedback mechanism perpetuating inflammatory responses in the heart that may lead to significant alterations in CV physiology and negatively affect systolic and/or diastolic functions of the heart, induce cardiac hypertrophy, and increase overall risk for CV disease development.

### Radiation and Aging

In our studies, proton irradiation did not negatively impact heart function 3 months post-IR, but showed negative effects 10 months post–IR. This was manifested by decreased LV systolic pressure and decreased dP/dt_min_, suggesting alterations in both contractile and relaxation functions in proton-irradiated hearts 10 months after initial exposure. There was also a considerable increased in cardiac fibrosis in proton-irradiated hearts at 10 months. In terms of molecular signaling there was an activation of NFATc4 in proton-irradiated hearts that may indicate activation of cardiac hypertrophy signaling. However, following ^56^Fe ion irradiation, significant negative effects on cardiac homeostasis and function in the heart were observed 3 months post-IR, but not at 10 months. In spite of increased cardiac fibrosis in ^56^Fe ion-irradiated hearts at 10 months, cardiac physiology functions were maintained and rather mildly improved at 10 months post-IR.

We evaluated key molecular pathways in post-IR hearts. Sodium-calcium exchanger (NCX) plays an essential role in Ca^2+^ handling by regulating Ca^2+^ transport [22]. A large body of evidence suggest that up-regulation of NCX is a common feature of HF [29], [30], however, increased NCX protein levels by itself do not corroborate conclusively with HF, as both beneficial and deleterious effects of NCX in heart failure have been proposed [29], [31]. At the same time, reduced expression and activity of SERCA2a has been also associated with HF and alterations in diastolic function [29], [31]. Our findings of increased NCX and SERCA2a levels at 1 month post-IR followed by a decrease at 3 months and unchanged expression in proton and increased expression in ^56^Fe ions by 10 months post-IR indicate that both charged particle types resulted in dysregulation of these important signaling proteins over 10 months.

One of the major mechanisms of cardiac compensation is hypertrophy of the heart and the transcription factor, NFATc4, is one of the well-studied regulators of cardiac hypertrophy [32], [33]. NFATc4 under basal unstimulated condition remains highly phosphorylated and inactive [23] and de-phosphorylation of NFATc4 results in its activation, which is associated with adult cardiac hypertrophy [33]. MAP kinases, such as p38 MAPK, have been shown to regulate NFATc4 phosphorylation and nuclear translocation [23]. This activation of p38 affects de-phosphorylated NFATc4 by re-phosphorylation that antagonizes the Ca^2+^-mediated de-phosphorylation and nuclear translocation of NFATc4, which results in NFATc4 being exported out of the nucleus and termination of NFATc4-mediated transcription [23], [32], [34], hence cardiac hypertrophy signaling. Meanwhile activation of the p38 MAPK pathway through phosphorylation of p38 also results in protection against heart failure by aiding in cardiac restoration [35]. Decreased activation of p38 MAPK signaling in ^56^Fe ion-irradiated hearts at 1, 3 and 10 months could further negate a possible cardiac protective mechanism(s). There was activation of NFATc4 in ^56^Fe ion-irradiated hearts that may indicate activation of cardiac hypertrophy signaling.

Taken together, our findings in *radiation and aging model* demonstrated that at the dose of 50 cGy protons induced initial cardiac hypertrophy followed by gradual deterioration of cardiac function over the course of 10 months post-IR. An approximately equitoxic dose (assuming an RBE of ∼3) of 15 cGy of 1 GeV/n ^56^Fe ions induced an early (1 and 3 months) onset of cardiac dysfunction. The early and considerable ^56^Fe ion-mediated negative CV developments may be indicative of increased risk of cardiac de-compensation, and may also be predictive of an increased risk of impaired responses to an ischemic event.

### Radiation, Aging and AMI

We evaluated the effect of low dose proton and ^56^Fe ion irradiation on the recovery of the heart in a well-accepted ischemic model of induced AMI (via ligation of left anterior descending carotid artery). At 50 cGy, 1 GeV a single dose of ^1^H-IR may have positive effects for post-ischemic recovery processes in AMI model, at any time point examined up to 10 months. These include significantly improved recovery of cardiac contractile and relaxation functions and enhanced cardiac remodeling. A number of molecular pathways that regulate cardiac contractility, survival, proliferation and angiogenesis may be responsible for these positive effects in ^1^H-IR hearts, over the period of 10 months. At 15 cGy, 1 GeV/n, a single dose of ^56^Fe-IR presents long-lasting negative effects for post-ischemic recovery and regeneration processes in AMI model, at any time point examined up 10 months. These include significant decreases in cardiac contractile and relaxation function, LV posterior wall hypertrophy and the worst cardiac remodeling. A number of molecular pathways that regulate cardiac contractility, survival, proliferation and angiogenesis may be responsible for these negative effects in ^56^Fe-IR hearts, over the period of 10 months.

To determine possible underlying mechanism involved in the long-lasting negative effects of ^56^Fe-IR and positive effects of ^1^H-IR on post-ischemic recovery we analyzed several molecules in the key signaling pathways of angiogenesis, survival, proliferation and apoptosis. The proper regulation of the expression and the activity of VEGF-A, Akt, Erk1/2 and MAPK p38 aids in recovery and repair after AMI and, ultimately, in the cardiac remodeling with the smallest possible scar tissue formation [36]. VEGF-A plays a critical role in the recovery of cardiac tissue following AMI to significantly improve heart function through greater cell proliferation and increased vascular densities in hearts when VEGF-A is present, helping cardiac tissue to restore normal functions after ischemic injury [25]. The significantly reduced VEGF-A levels in ^56^Fe-IR hearts by 3 days post-AMI (3 month post-IR) indicate decreased angiogenic signaling due to ^56^Fe-IR, when compared to non-IR+AMI and to lesser degree to ^1^H-IR+AMI hearts.

Two well-characterized signaling protein kinases, Erk1/2 and Akt, are expressed in the heart following AMI to improve and restore heart function [37]. Erk1/2 is an important regulator of inflammation and apoptosis [37], which is essential for normal healing process and removal of damage. Akt also has a role in the survival signaling and reduction of inflammation that occurs following AMI [26]. Reduced expression of either or both of these proteins in combination, as seen in ^56^Fe-IR+AMI hearts by 3 days (3 month post-IR) can have deleterious effects on post-AMI recovery since both are required for removal of ischemia induced damage and cell survival. In addition to Erk1/2, another member of mitogen-activated protein kinase cascade p38 MAPK, was significantly decreased in ^56^Fe-IR+AMI compared to control+AMI and ^1^H-IR+AMI hearts. In cardiac cells p38 MAPK functions as a stress response and a survival gene and activation of p38 signaling has been reported to prevent post-AMI heart failure through up-regulation of angiogenesis and activation of anti-apoptotic mechanisms [27]. The undetectable levels of phosphorylated p38 (p-p38) in ^56^Fe-IR+AMI hearts 3 months post-IR and 3 days after AMI underscore further impairment of cardiac protection signaling and suggest an imminent development of HF in ^56^Fe-IR hearts [28].

Since it is immeasurably important to determine possible factors that may increase CV degenerative risks due to low dose ionizing particle radiation that will allow for development of mitigating factors to reduce excess CV risks and to maintain cardiac homeostasis after IR exposure we would like to summarize our findings as follows: *(1)* whether it is ^56^Fe- or ^1^H-IR, the heart shows stress and signs of attempted restoration to normal cardiac functioning; *(2)* single low dose full body ^56^Fe and ^1^H-IR induced effects on myocardium are of long duration; *(3)* the significant negative effects on systolic and diastolic heart functions post-IR, is largely associated with decreased Ca^2+^ handling, increased hypertrophy signaling, decreased cardiac contractile signaling and decreased p38 MAPK signaling; *(4)* it is likely that significant alterations in Ca^2+^ (NCX, SERCA2a) and cardiac hypertrophy signaling (NFATc4, p38 MAPK) detected in our longitudinal studies are early responses to cardiac stress [38], that may arise from the excessive demand on the heart due to prolonged activation of compensatory mechanisms; this could lead to changes in gene transcription and metabolism, thus representing part of the vicious circle that may precipitate HF; *(5)* in the ischemic model of recovery after AMI, multiple signaling pathways that regulate angiogenesis, survival, proliferation and protein synthesis are significantly inhibited in ^56^Fe-IR and activated in ^1^H-IR hearts 3 months post-IR and 3 days post-AMI, while by in 10 months post-IR and 3 days post-AMI the expression of these genes were increased.

Taken together, our data suggest that low dose exposures of charged particles present in the space radiation environment may have significant impact on the CV system. These results are the first reports of the effects of single, low-dose, whole-body proton and ^56^Fe ion irradiation on the CV system during normal aging and under ischemic conditions. We believe that these findings will provide useful information for risk analysis efforts for future NASA exploration-type space missions and aid in the development of mitigating countermeasures to reduce CV risks during long-duration deep-space missions. In addition, by 2012, more than 120,000 cancer patients in 16 counties [15] have been treated using particle radiation therapy (RT), primarily protons but also including carbon and other HZE ions, with similar centers being planned and constructed every year. Our studies may also provide a foundation for the development of therapeutic measures to prevent CV morbidity and mortality due to cancer radiotherapy (conventional and/or the particle), as well as accidental and occupational IR exposures.

## Supporting Information

Figure S1 **Representative ×100 confocal microscopy images for triple immunostaining with** ***p*** **-H2AX (green), endothelial cell (EC) marker Isolectin/B4 (red) and Topro-3 nuclear staining (blue) in heart tissue after a single, low-dose, full-body (A) ^1^H-IR and (B) ^56^Fe-IR mice at 2, 5, 24 hours, and 7, 14 and 28 days post-IR along with respective non-IR control.** Green staining within Topro-3 stained nuclei (blue) indicates *p*-H2AX positive foci.
(TIF)

Figure S2 **Representative ×200 confocal microscopy images for immunostaining with macrophage marker – CD68 (green) and Topro-3 stained nuclei (red) in heart tissue after a single, low-dose, full-body (A) ^1^H-IR mice and (B) ^56^Fe-IR mice at 2, 5, 24 hours, and 7, 14, 28 days, 2 and 3 months post-IR along with respective non-IR control.** Note, when CD68-green cytoplasmic staining is overlaid with nuclei-red staining it appears as yellow/green staining.
(TIF)

Figure S3 **Representative ×200 brightfield microscopy images for Masson's trichrome staining in non-IR control, ^1^H-IR and ^56^Fe-IR mice hearts at (A) 1 month post-IR; (D) 3 months post-IR; (G) 10 months post-IR.** In top row images for 1, 3 and 10 month time points blue staining indicates fibrosis; (**B, E and H**), Middle row are the same representative images of the top raw where white spaces between muscle fibers digitally filled with black color to eliminate the histological artifact spaces to allow for error free analysis of the heart tissue fibrosis using computer-assisted algorithm and Image J program; (**C, F and I**), Representative Image J algorithm generated images (bottom row) of the same respective non-IR control, ^1^H-IR and ^56^Fe-IR images in (**A, D, G and B, E, H**).
(TIF)

Figure S4 (**A**) Representative ×100 confocal microscopy image for immunostaining with cTnI (red) and Topro-3-stained nuclei (blue) in full-body ^56^Fe-IR mouse heart tissue at 3 months post-IR and 3 days after AMI to demonstrate initial infarct tissue region (within yellow dotted line), the border zone and normal heart tissue region. (**B**) Graphic representation of initial infarct size (µm^2^) by day 3 post-AMI in the hearts of ^1^H-IR, ^56^Fe-IR and non-IR control mice 3 months post-IR; *n* = 3 treatment group, *P* = NS, between all groups.
(TIF)

Figure S5 **Representative ×100 brightfield microscopy collaged images of non-IR control, ^1^H-IR and ^56^Fe-IR mice heart at: (A) 1 month post-IR, (B) 3 months post-IR and (C) 10 months post-IR stained with Hematoxylin and Eosin (H&E).** Dashed lines in each representative image denotes infarct region at 28 days post-AMI for 1, 3 and 10 month time points in all three treatment groups.
(TIF)

Figure S6 **Representative ×100 brightfield microscopy collaged images at 1 month time point for (A) non-IR control, (B) ^1^H-IR and (C) ^56^Fe-IR for 5 animals (biological replicates)/treatment group stained with Masson's trichrome to demonstrate cardiac remodeling post-AMI at day 28 and the infarct region (stained in blue).**
(TIF)

Figure S7 **Representative ×100 brightfield microscopy collaged images at 3 month time point for (A) non-IR control, (B) ^1^H-IR and (C) ^56^Fe-IR for 5 animals (biological replicates)/treatment group stained with Masson's trichrome to demonstrate cardiac remodeling post-AMI at day 28 and the infarct region (stained in blue).**
(TIF)

Figure S8 **Representative ×100 brightfield microscopy collaged images at 10 month time point for (A) non-IR control, (B) ^1^H-IR and (C) ^56^Fe-IR for 4 animals (biological replicates)/treatment group stained with Masson's trichrome to demonstrate cardiac remodeling post-AMI at day 28 and the infarct region (stained in blue).**
(TIF)
