# Correction: Cardiovascular Risks Associated with Low Dose Ionizing Particle Radiation

**DOI:** 10.1371/journal.pone.0142764

**Published:** 2015-11-06

**Authors:** Xinhua Yan, Sharath P. Sasi, Hannah Gee, JuYong Lee, Yongyao Yang, Raman Mehrzad, Jillian Onufrak, Jin Song, Heiko Enderling, Akhil Agarwal, Layla Rahimi, James Morgan, Paul F. Wilson, Joseph Carrozza, Kenneth Walsh, Raj Kishore, David A. Goukassian

In the Funding section, the grant number from the funder National Aeronautic and Space Administration has been updated by the funder. The updated grant number is: NNX11AD22G.
