# Supplementary figures and images for: Cardiovascular Risks Associated with Low Dose Ionizing Particle Radiation
Source: PLoS One. 2014 Oct 22;9(10):e110269. doi: 10.1371/journal.pone.0110269 (PMC4206415; doi:10.1371/journal.pone.0110269)

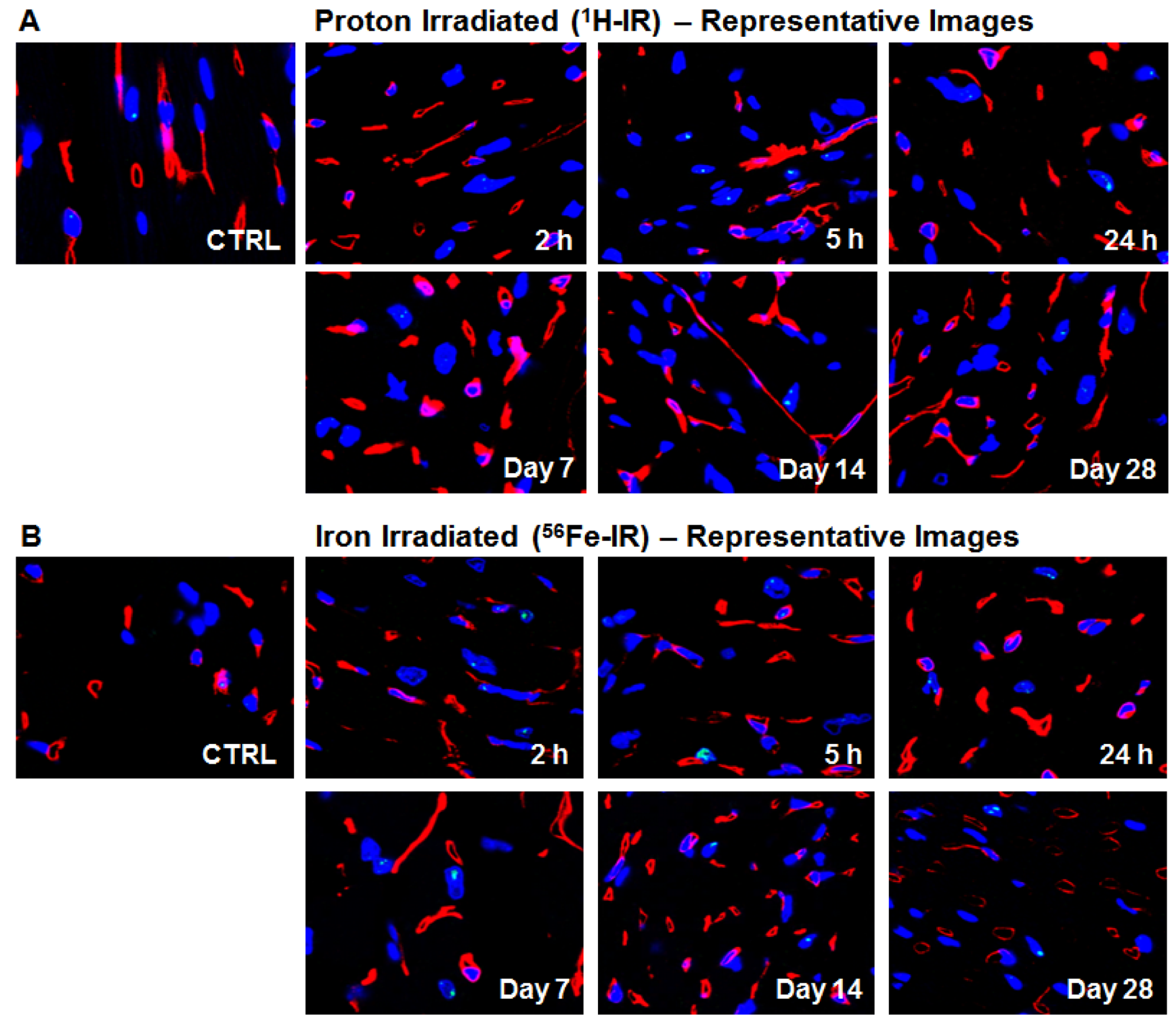

Supplement: Figure S1 — Representative ×100 confocal microscopy images for triple immunostaining with p -H2AX (green), endothelial cell (EC) marker Isolectin/B4 (red) and Topro-3 nuclear staining (blue) in heart tissue after a single, low-dose, full-body (A) 1H-IR and (B) 56Fe-IR mice at 2, 5, 24 hours, and 7, 14 and 28 days post-IR along with respective non-IR control. Green staining within Topro-3 stained nuclei (blue) indicates p-H2AX positive foci. (TIF) [file pone.0110269.s001.tif]

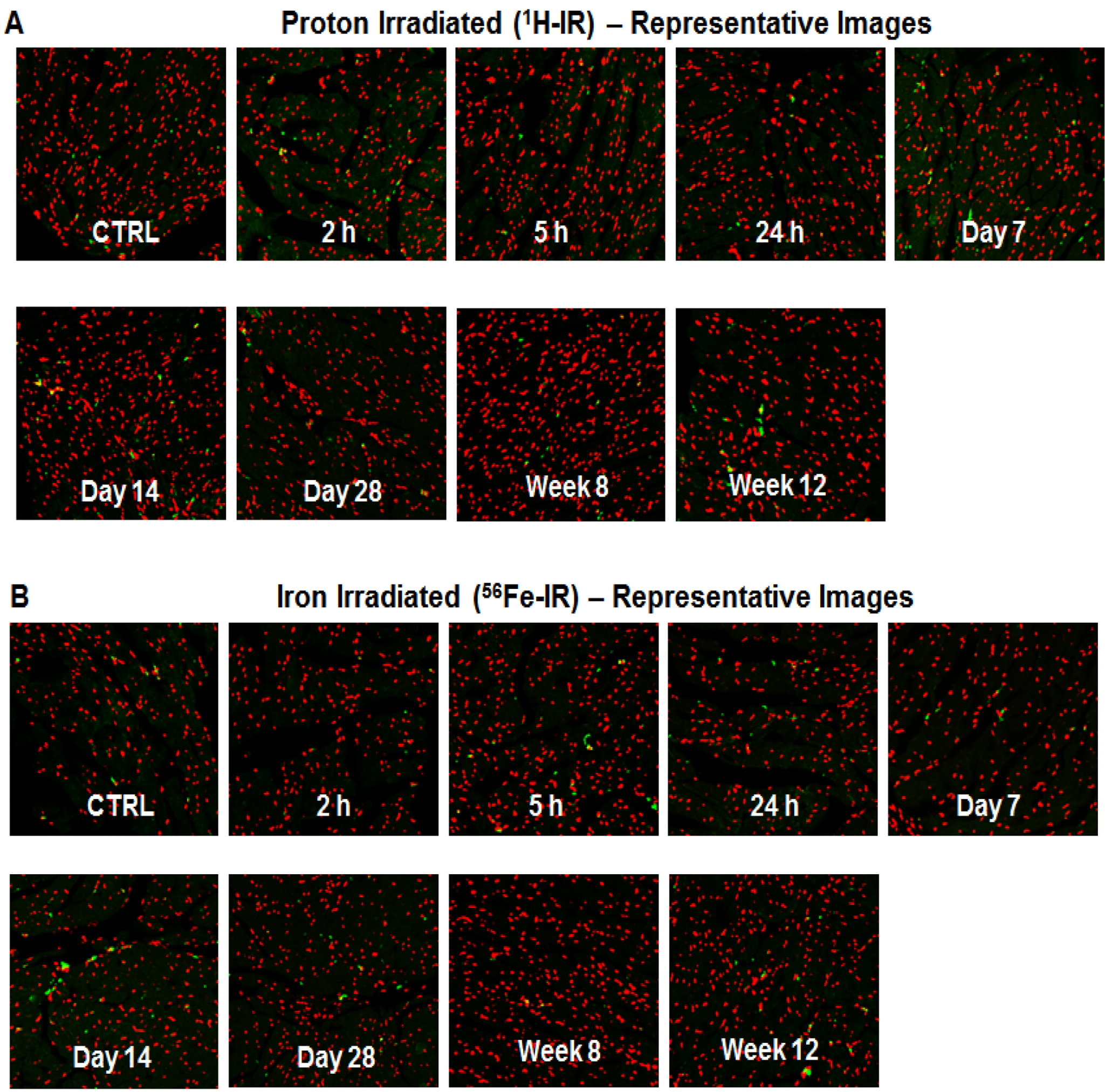

Supplement: Figure S2 — Representative ×200 confocal microscopy images for immunostaining with macrophage marker – CD68 (green) and Topro-3 stained nuclei (red) in heart tissue after a single, low-dose, full-body (A) 1H-IR mice and (B) 56Fe-IR mice at 2, 5, 24 hours, and 7, 14, 28 days, 2 and 3 months post-IR along with respective non-IR control. Note, when CD68-green cytoplasmic staining is overlaid with nuclei-red staining it appears as yellow/green staining. (TIF) [file pone.0110269.s002.tif]

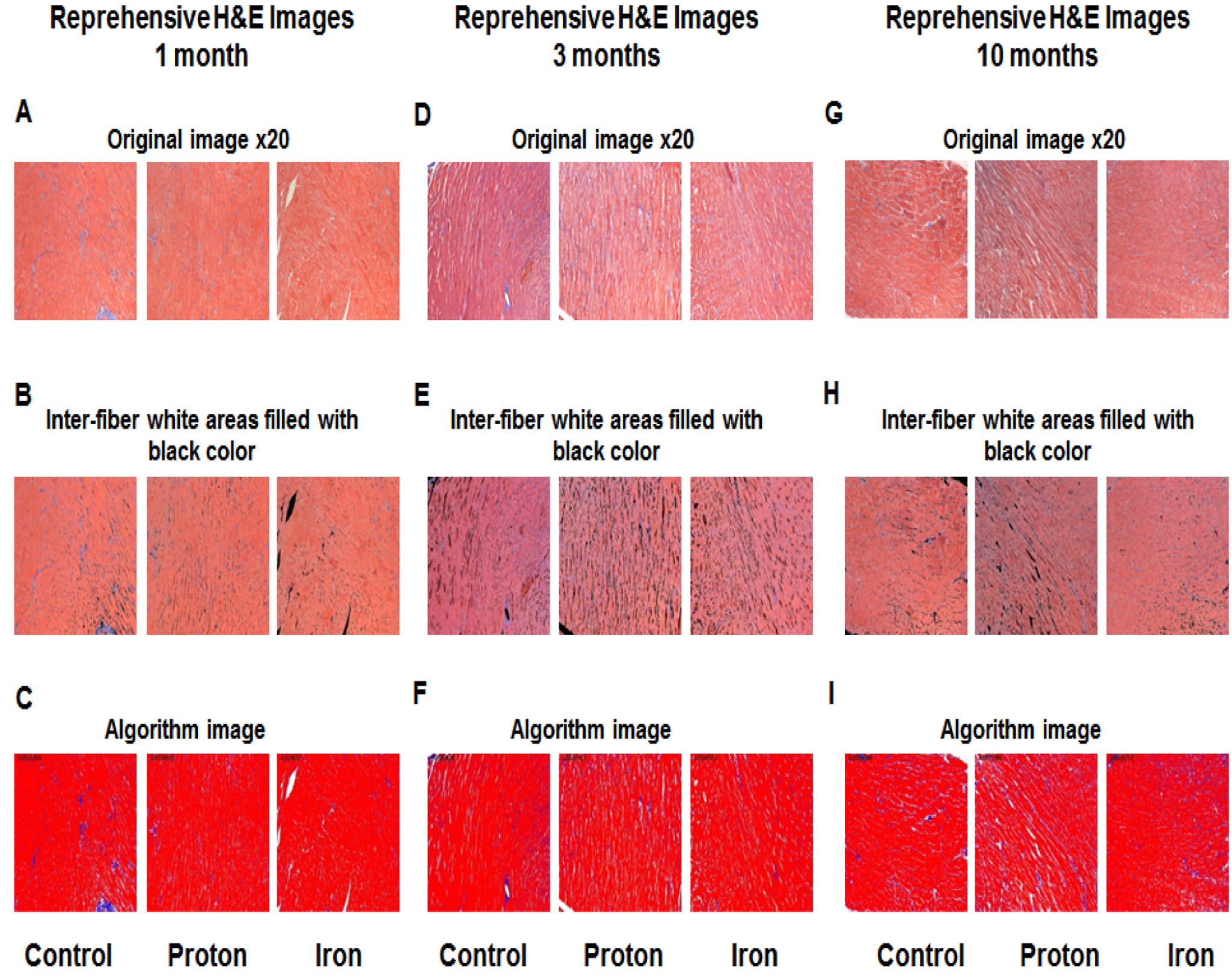

Supplement: Figure S3 — Representative ×200 brightfield microscopy images for Masson's trichrome staining in non-IR control, 1H-IR and 56Fe-IR mice hearts at (A) 1 month post-IR; (D) 3 months post-IR; (G) 10 months post-IR. In top row images for 1, 3 and 10 month time points blue staining indicates fibrosis; (B, E and H), Middle row are the same representative images of the top raw where white spaces between muscle fibers digitally filled with black color to eliminate the histological artifact spaces to allow for error free analysis of the heart tissue fibrosis using computer-assisted algorithm and Image J program; (C, F and I), Representative Image J algorithm generated images (bottom row) of the same respective non-IR control, 1H-IR and 56Fe-IR images in (A, D, G and B, E, H). (TIF) [file pone.0110269.s003.tif]

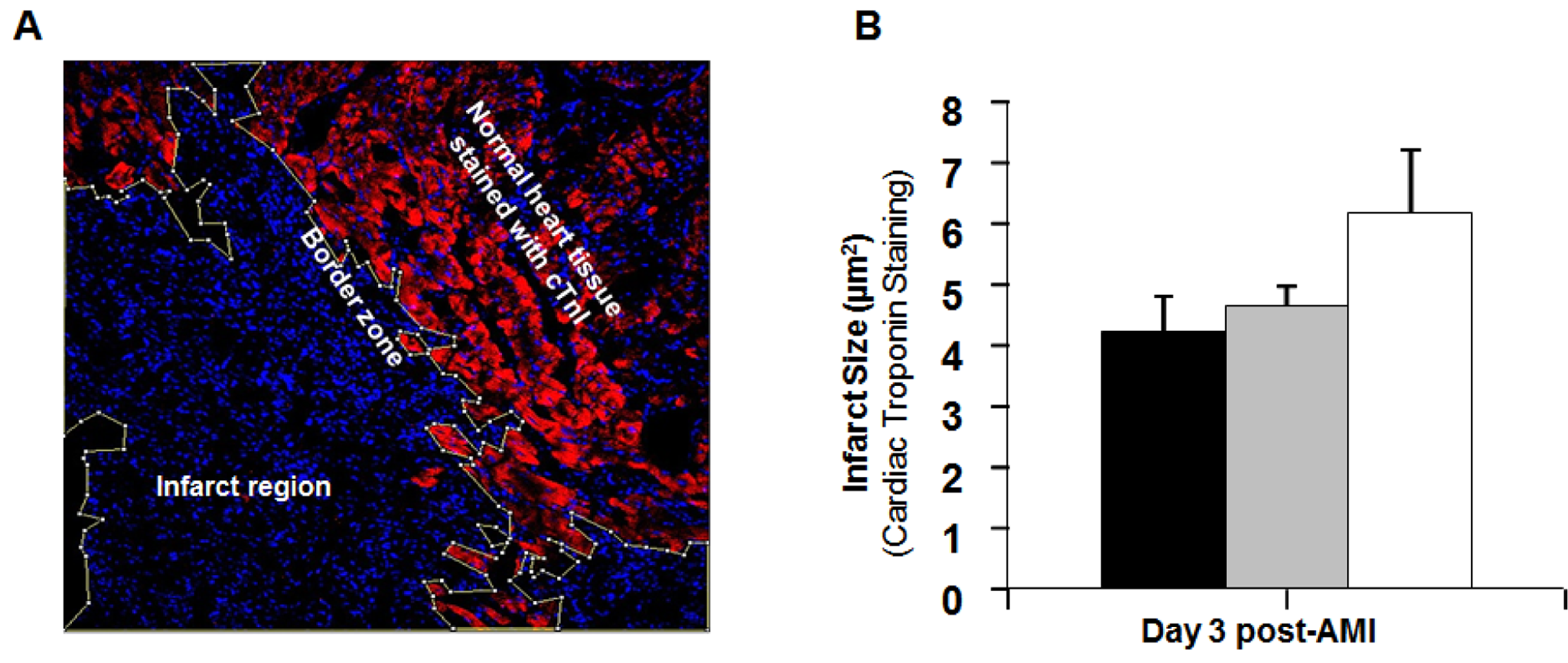

Supplement: Figure S4 — (A) Representative ×100 confocal microscopy image for immunostaining with cTnI (red) and Topro-3-stained nuclei (blue) in full-body 56Fe-IR mouse heart tissue at 3 months post-IR and 3 days after AMI to demonstrate initial infarct tissue region (within yellow dotted line), the border zone and normal heart tissue region. (B) Graphic representation of initial infarct size (µm2) by day 3 post-AMI in the hearts of 1H-IR, 56Fe-IR and non-IR control mice 3 months post-IR; n = 3 treatment group, P = NS, between all groups. (TIF) [file pone.0110269.s004.tif]

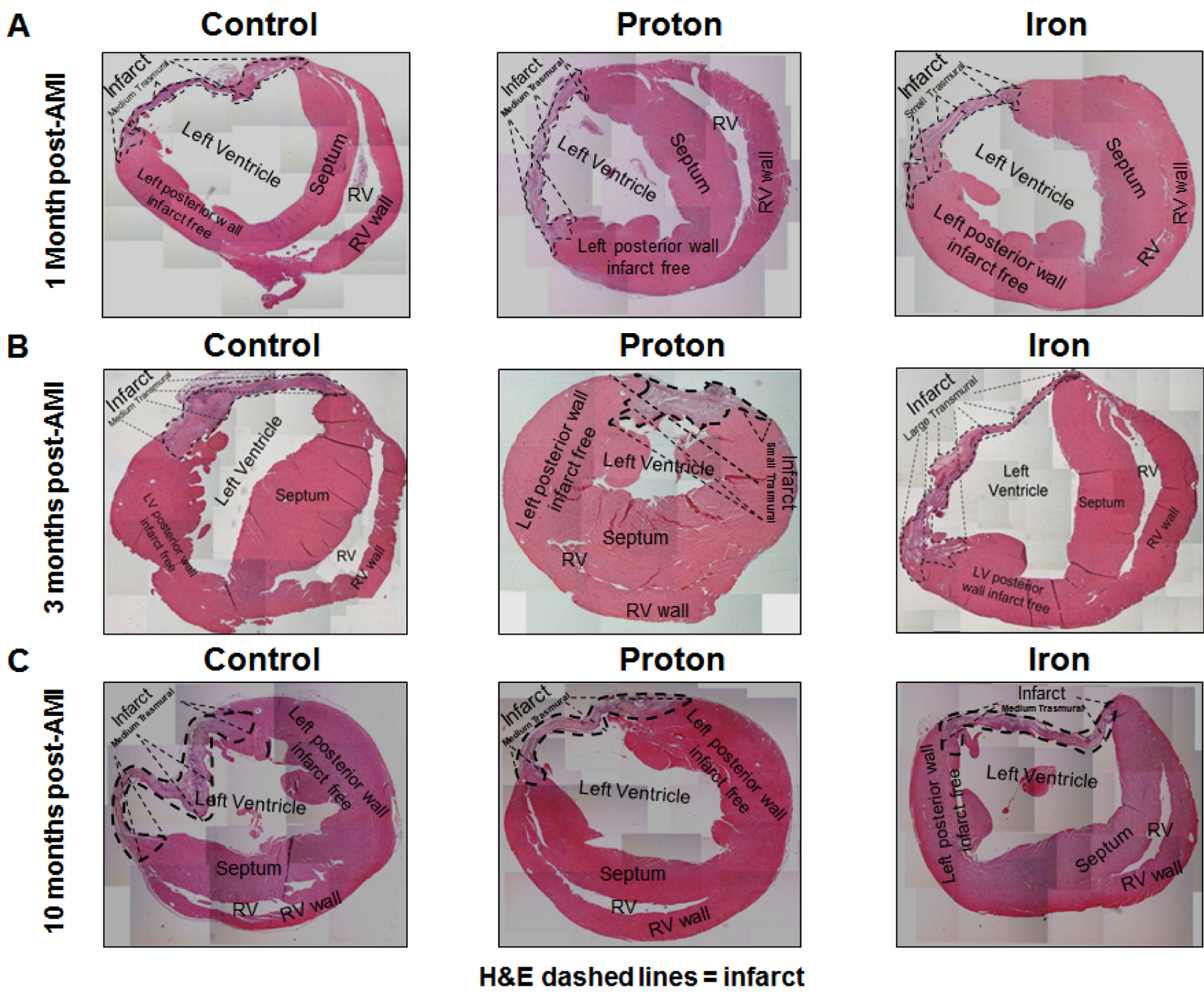

Supplement: Figure S5 — Representative ×100 brightfield microscopy collaged images of non-IR control, 1H-IR and 56Fe-IR mice heart at: (A) 1 month post-IR, (B) 3 months post-IR and (C) 10 months post-IR stained with Hematoxylin and Eosin (H&E). Dashed lines in each representative image denotes infarct region at 28 days post-AMI for 1, 3 and 10 month time points in all three treatment groups. (TIF) [file pone.0110269.s005.tif]

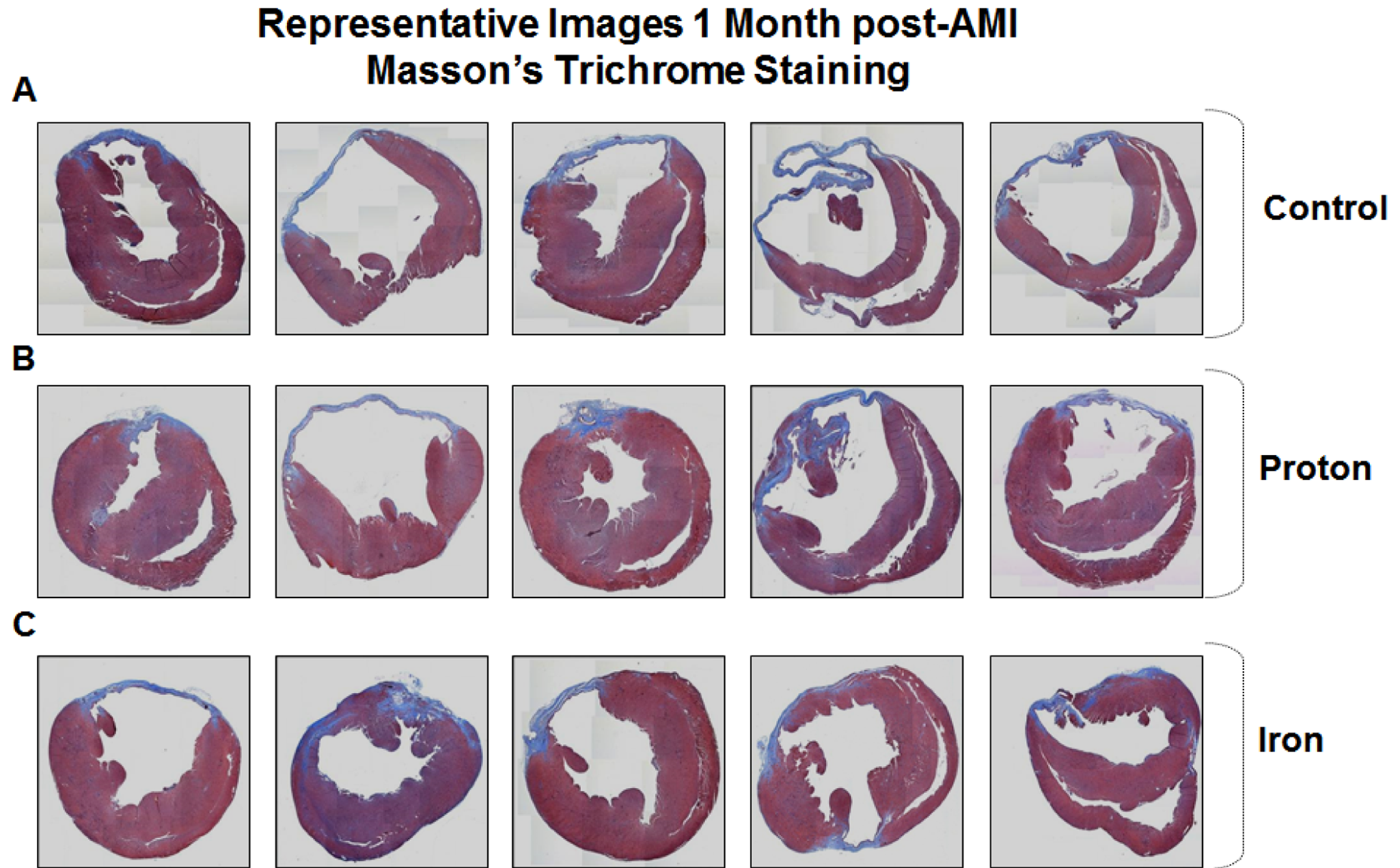

Supplement: Figure S6 — Representative ×100 brightfield microscopy collaged images at 1 month time point for (A) non-IR control, (B) 1H-IR and (C) 56Fe-IR for 5 animals (biological replicates)/treatment group stained with Masson's trichrome to demonstrate cardiac remodeling post-AMI at day 28 and the infarct region (stained in blue). (TIF) [file pone.0110269.s006.tif]

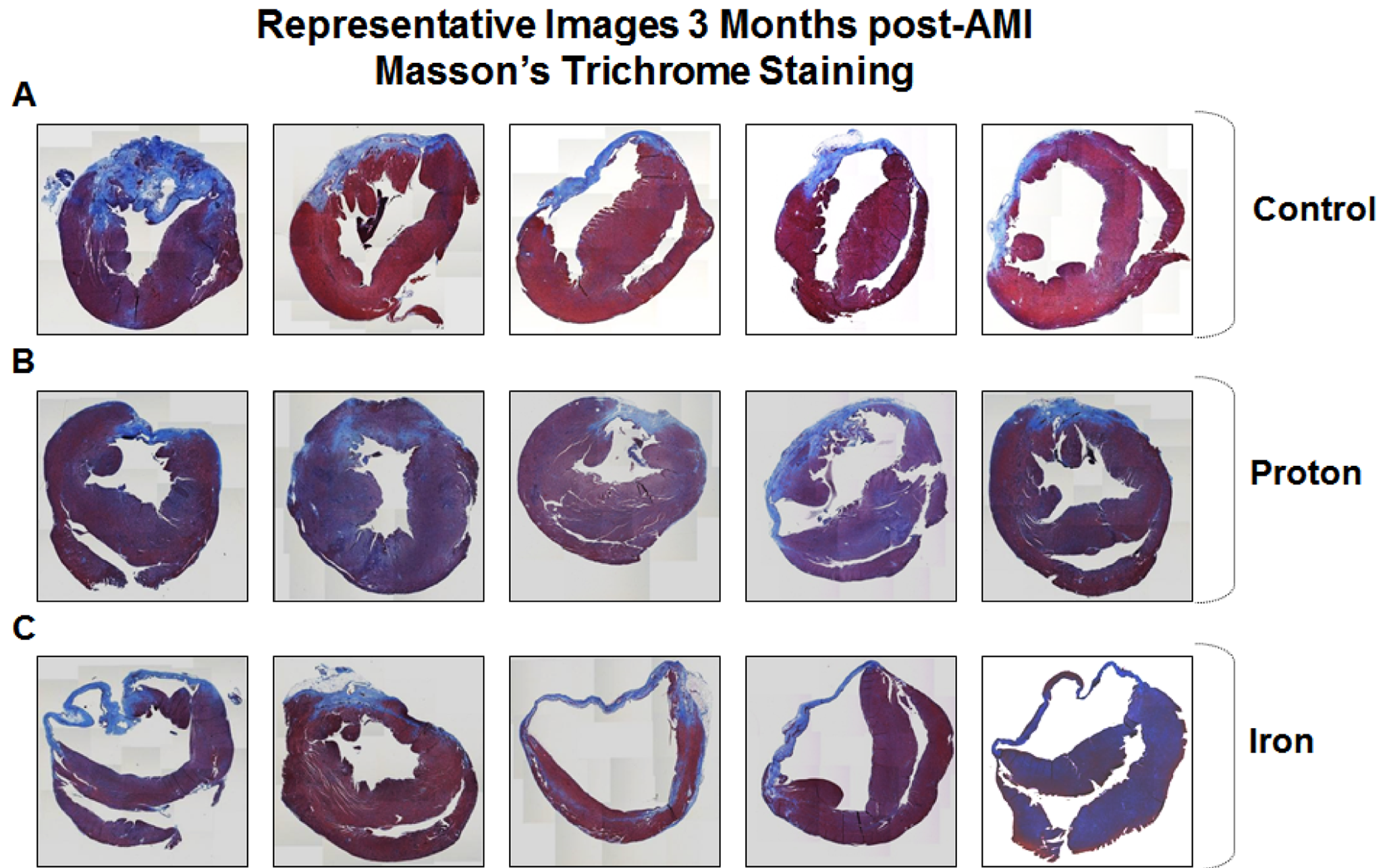

Supplement: Figure S7 — Representative ×100 brightfield microscopy collaged images at 3 month time point for (A) non-IR control, (B) 1H-IR and (C) 56Fe-IR for 5 animals (biological replicates)/treatment group stained with Masson's trichrome to demonstrate cardiac remodeling post-AMI at day 28 and the infarct region (stained in blue). (TIF) [file pone.0110269.s007.tif]

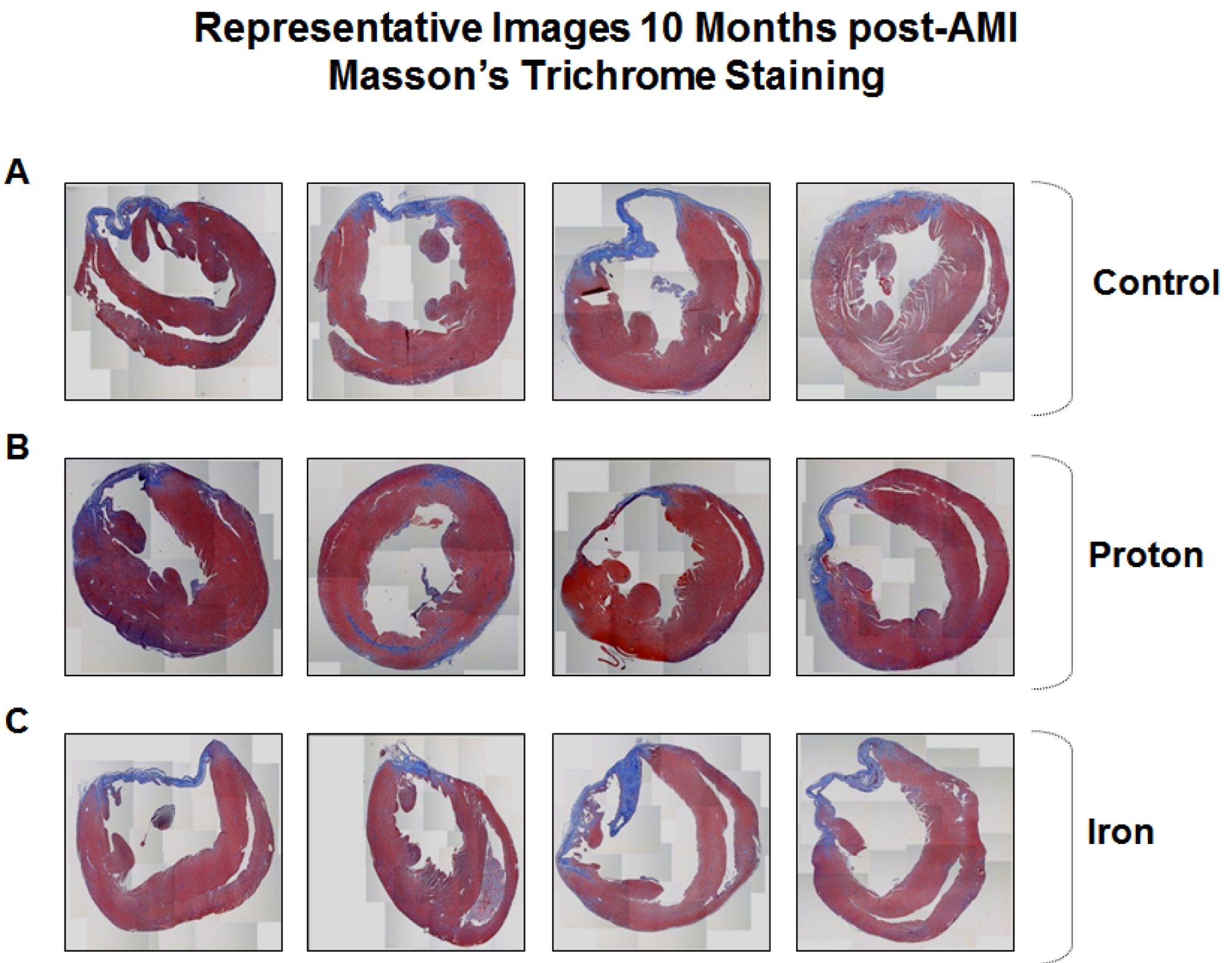

Supplement: Figure S8 — Representative ×100 brightfield microscopy collaged images at 10 month time point for (A) non-IR control, (B) 1H-IR and (C) 56Fe-IR for 4 animals (biological replicates)/treatment group stained with Masson's trichrome to demonstrate cardiac remodeling post-AMI at day 28 and the infarct region (stained in blue). (TIF) [file pone.0110269.s008.tif]
